# The male-type mitochondrial genome of the freshwater mussel *Potamilus streckersoni* Smith, Johnson, Inoue, Doyle, & Randklev, 2019 (Bivalvia: Unionidae)

**DOI:** 10.1080/23802359.2022.2134750

**Published:** 2022-10-27

**Authors:** Raquel Mejia-Trujillo, Chase H. Smith

**Affiliations:** Department of Integrative Biology, University of Texas, Austin, TX, USA

**Keywords:** Doubly uniparental inheritance, Unionida, Ambleminae

## Abstract

The global decline of freshwater mussels emphasizes the need to establish genetic resources to better understand their biology, including a unique mitochondrial biology known as doubly uniparental inheritance. In this study, we present the complete male-type (M-type) mitochondrial genome of the freshwater mussel, *Potamilus streckersoni* Smith, Johnson, Inoue, Doyle, & Randklev, 2019. The M-type mtDNA is approximately 16 kilobases and contains 22 tRNAs, two rRNAs, and 14 protein-coding genes, including a male-specific open reading frame. Read coverage revealed that M-type mtDNA was more abundant than female-type mtDNA in male gonadal tissue, with respect to a non-spawning male individual. Novel mitogenomes were resolved within previously described sex-specific monophyletic clades across the subfamily Ambleminae. The availability of high-quality nuclear and mitochondrial genomic data for *P. streckersoni* makes it a model for future research into the potential role of mtDNA in sex determination or sexual development in freshwater mussels.

## Introduction

Freshwater mussels (Bivalvia: Unionidae) are aquatic mollusks concentrated in North America (Graf and Cummings 2021), with approximately 300 species in the United States and Canada (Williams et al. 2017). Unfortunately, systemic declines have led the group to be considered one of the most imperiled in North America (Strayer et al. 2004; Haag and Williams 2014), which is problematic considering freshwater mussels perform essential functional roles in freshwater ecosystems (Vaughn and Hakenkamp 2001). Recent declines have invoked a need for additional research (Ferreira-Rodríguez et al. 2019), which includes the establishment of genetic resources to facilitate the research of freshwater mussel biology.

Several bivalve lineages, including freshwater mussels, possess a unique mitochondrial inheritance pattern known as doubly uniparental inheritance (DUI), which involves the transmission of sex-specific mtDNAs (Hoeh et al. 1991; Breton et al. 2007; Zouros 2013). Females are homoplasmic for a female-type (F-type) mtDNA that is transmitted to all offspring (Zouros 2013), similar to organisms with strictly maternal mitochondrial inheritance. In males, however, male-type (M-type) mtDNA is transmitted from fathers only to their sons. In freshwater mussels, F- and M-type mtDNAs are highly distinctive, differing by more than 50% of their amino acid sequences (Breton et al. 2007). Freshwater mussel mtDNAs also contain haplotype-specific open reading frames (ORFs) that have no homology to known genes. A female ORF (F-ORF) has been detected in F-type mtDNA, while a male ORF (M-ORF) has been detected in M-type mtDNA (Breton et al. 2011). Given this, an association between nuclear gene products and mtDNA ORFs is speculated to play a role in sex determination (Breton et al. 2022), but the lack of genetic resources has hindered research into these topics (e.g. Renaut et al. 2018; Capt et al. 2019).

**Figure 1.**
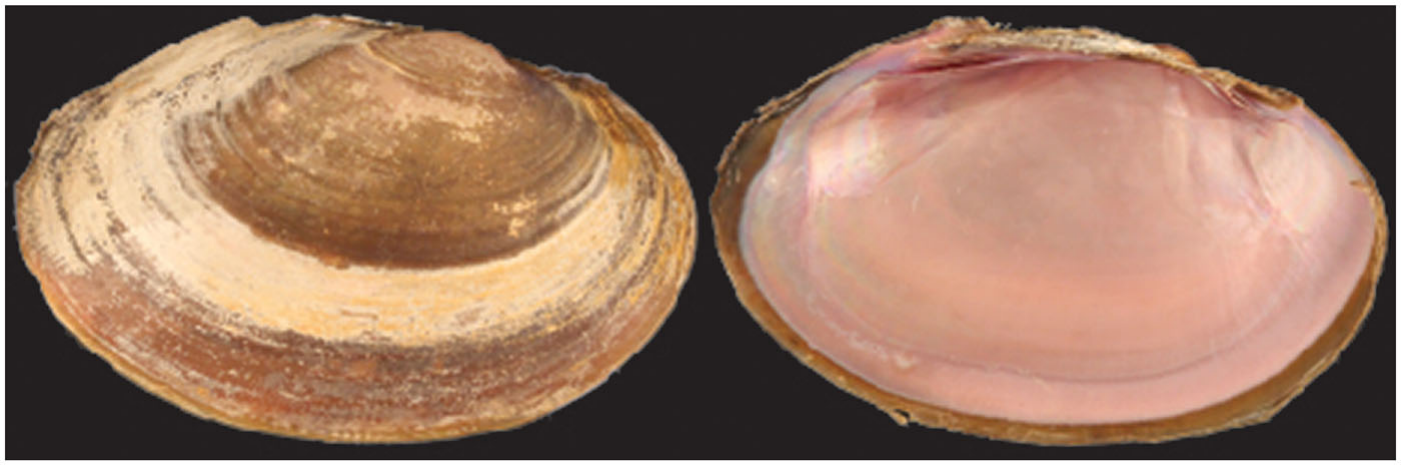
*Potamilus streckersoni* holotype (UF439497) designated by Smith et al. (2019).

*Potamilus streckersoni* Smith, Johnson, Inoue, Doyle, & Randklev, 2019 (Figure 1) is one of the few freshwater mussel species with a high-quality *de novo* nuclear genome assembly (Smith 2021). In this study, we assemble the M-type mtDNA of *P. streckersoni*, which completes the availability of genetic resources for the species. With reference F-type mtDNA, M-type mtDNA, and nuclear genome assemblies available, *P. streckersoni* represents a model for investigating the biological function of DUI and mitonuclear ecology in freshwater mussels.

## Methods

One adult male *P. streckersoni* was collected from the type locality in the Brazos River drainage (Latitude: 30.86586° N, Longitude: 96.69575° W) under Scientific Research Permit SPR-0717-156 issued by the Texas Parks and Wildlife Department. The specimen was deposited in the Joseph Britton Freshwater Mussel Collection (Charles Randklev; Charles.Randklev@ag.tamu.edu) under voucher number 10001.6. DNA was extracted from gonadal tissue using the Qiagen PureGene Kit (Hilden, Germany) with the manufacturer’s recommended protocol. High-molecular weight DNA was ensured by visualizing the isolation on a 1% agarose gel stained with GelGreen^®^ nucleic acid stain (Biotium, Hayward, CA). DNA quantity and quality was assessed using a Qubit™ fluorometer and a NanoDrop™ One (ThermoFisher Scientific, Waltham, MA), respectively. A genomic DNA library was constructed with the NEBNext Ultra™ II Library Prep Kit (Ipswich, MA) using standard protocols. The resulting library was sequenced using 100-bp single end reads on an Illumina NovaSeq 6000 (San Diego, CA).

TRIM GALORE! v 0.6.7 (Krueger et al. 2021) was used to trim adaptor sequences and filter reads of less than 30 bases. F- and M-type mtDNAs were *de novo* assembled from the trimmed reads using MitoFinder v 1.4 (Allio et al. 2020). Gene limits for the M-type mtDNA were constructed using MITOS2 (Donath et al. 2019) and manually curated when needed. The M-ORF was manually annotated using Geneious v 2022.1.1 (Kearse et al. 2012). Final M-type tRNA gene limits were assessed using ARWEN v 1.2 (Laslett and Canbäck 2008) and manually curated when needed.

We investigated start codon variation by compiling start codons of all available Ambleminae mtDNAs in 14 PCGs, including the haplotype-specific ORFs when available. Considering both F- and M-type mtDNAs are present in gonadal tissue, we also compared relative read depth of each type. We mapped trimmed reads to the final assemblies using bwa-mem2 v 2.2.1 (Vasimuddin et al. 2019) and calculated average read depth using SAMtools v 1.15.1 (Li et al. 2009).

**Figure 2.**
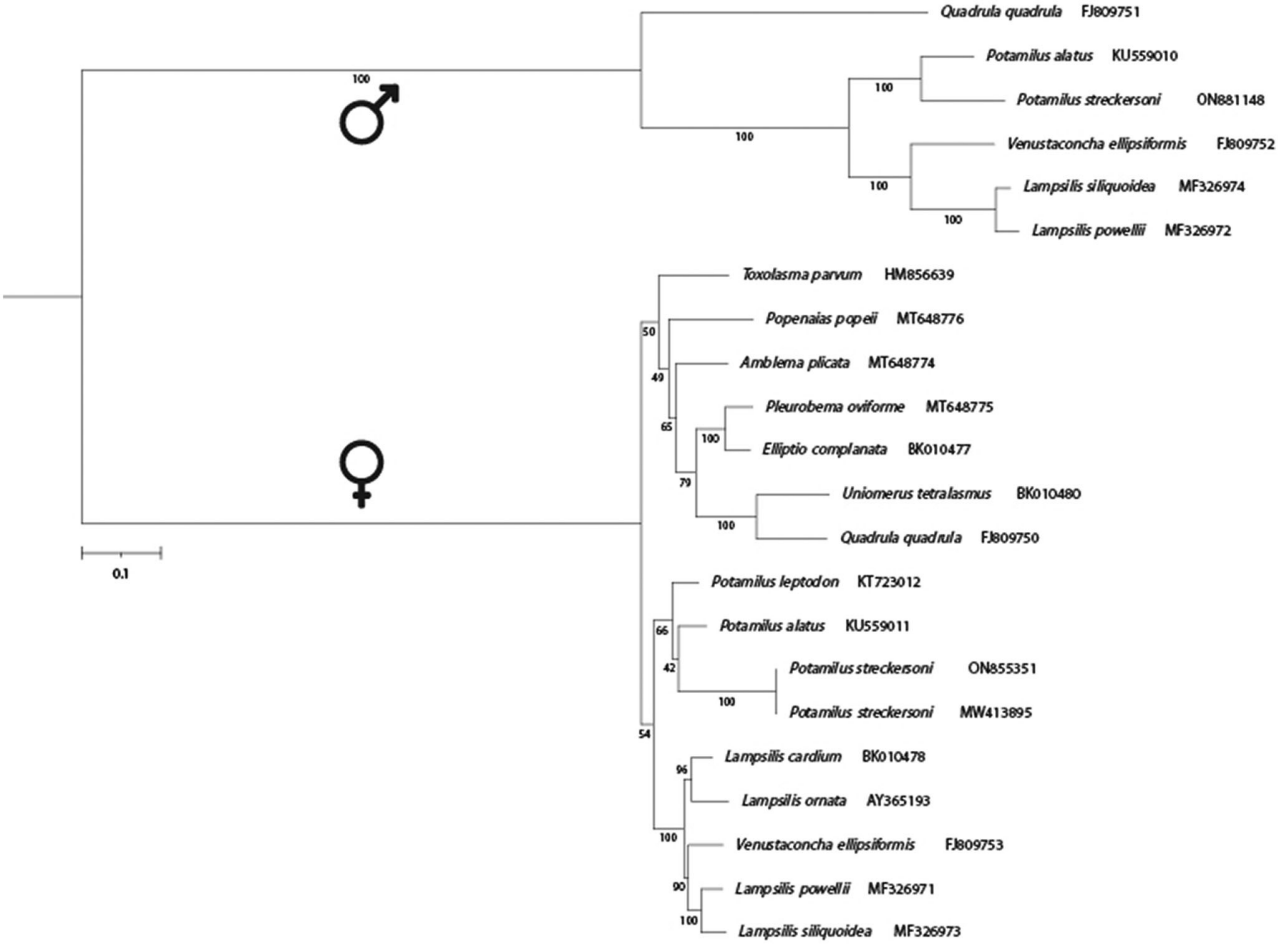
A multi-gene phylogenetic reconstruction of translated female- and male-type mitochondrial genes involved in oxidative phosphorylation. Associated GenBank accessions are included adjacent to each species in the subfamily Ambleminae. Ultrafast bootstrap support is referenced on nodes, where values greater than or equal to 95 represent strong support.

Annotations for 13 PCGs (excluding haplotype-specific ORFs) were extracted from 15 F- and five M-type mtDNAs (Figure 2) from NCBI and the novel *P. streckersoni* F- and M-type assemblies. PCGs were aligned using MACSE v 2 (Ranwez et al. 2011), and the resulting alignments were trimmed using trimAl v 1.4 (Capella-Gutierrez et al. 2009) with a gap threshold of 50%. Phylogenetic inference was performed on an amino acid alignment of 13 PCGs using IQ-TREE V 2.1.4 (Minh et al. 2020). ModelFinder (Kalyaanamoorthy et al. 2017) was used to select the best invertebrate mitochondrial model of amino acid evolution and 1000 ultrafast bootstrap replicates were used to assess nodal support (Hoang et al. 2018).

## Results and discussion

**Figure 3.**
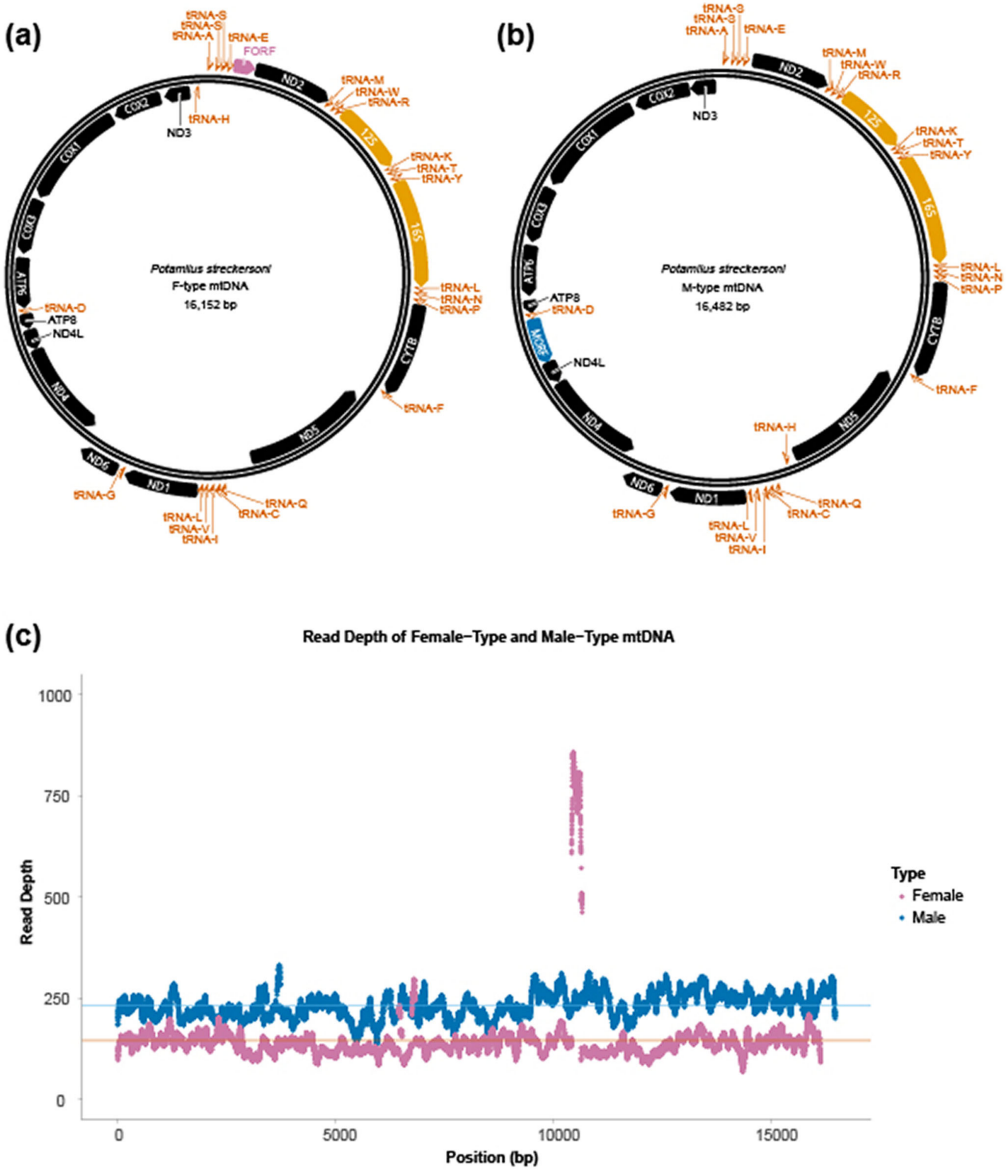
Female- (F-type) (a) and male-type (M-type) (b) mitogenome assemblies for *P. streckersoni*. Coloration of annotated features is as follows: protein-coding oxidative phosphorylation genes are represented in black, tRNAs are represented in dark orange, rRNAs are represented in light orange, and haplotype-specific open reading frames (ORFs) are represented in pink (F-ORF) and blue (M-ORF). Per base read depth statistics for F- and M-type mtDNA (c). Each blue (M-type) and pink (F-type) point represents coverage at a specific site. Lines represent the average value across each mtDNA.

Illumina sequencing generated approximately 70 million 100-bp single end reads. We were able to assemble both F- and M-type mtDNAs from a single male gonadal sample (GenBank accession nos. ON855351 and ON881148). The F-type assembly was 16,152 bp and similar, but smaller in size, than the previously published *P. streckersoni* F-type assembly that reported 16,293 bases (GenBank accession no. MW413895; Smith 2021). The M-type assembly was 16,482 bases and similar in size to the closely related species *Potamilus alatus* (Say, 1817), which was reported to be 16,560 bases (GenBank accession no. KU559010; Wen et al. 2017). Our annotations were consistent with annotations from available Ambleminae mitogenomes (Table 1). Each of the haplotype assemblies consisted of 22 tRNAs, two rRNAs, and 14 PCGs, which includes a haplotype-specific ORF (Figure 3). Both haplotypes utilize alternative start codons consistent with the invertebrate mitochondrial genetic code. Additionally, there are haplotype-specific start codon biases across *COX1*, *COX3*, *ND2*, *ND4*, and the ORFs (Table 1).

**Table 1.** Start codons based on the invertebrate mitochondrial translation code for annotated genes in female- (F) and male-type (M) mitogenomes examined in this study.

Taxon	Accession	Type	*ATP6*	*ATP8*	*COX1*	*COX2*	*COX3*	*CYTB*	*ND1*	*ND2*	*ND3*	*ND4*	*ND4L*	*ND5*	*ND6*	ORF
*Amblema plicata*	MT648774	♀	M	V	L	M	M	I	I	M	M	I	V	M	I	–
*Elliptio complanata*	BK010477	♀	M	–	L	M	M	I	I	M	M	I	M	M	I	–
*Lampsilis cardium*	BK010478	♀	M	M	L	M	M	I	I	M	M	I	M	M	I	–
*Lampsilis ornata*	AY365193	♀	M	M	L	M	M	I	I	M	M	I	V	M	I	–
*Lampsilis powellii*	MF326971	♀	M	M	L	M	M	I	I	M	M	I	V	M	I	L
*Lampsilis siliquoidea*	MF326973	♀	M	M	L	M	M	I	I	M	M	I	V	M	I	L
*Pleurobema oviforme*	MT648775	♀	M	V	L	M	M	I	I	M	M	I	M	M	I	–
*Popenaias popeii*	MT648776	♀	M	V	L	M	M	I	I	M	M	I	V	M	I	–
*Potamilus alatus*	KU559011	♀	M	V	I	M	M	I	I	M	M	I	V	M	M	I
*Potamilus leptodon*	KT723012	♀	M	V	L	M	M	I	I	M	M	I	M	I	I	–
*Potamilus streckersoni*	MW413895	♀	M	V	I	M	M	I	I	M	M	I	V	V	I	I
*Potamilus streckersoni*	ON855351	♀	M	V	I	M	M	I	I	M	M	I	V	V	I	I
*Quadrula quadrula*	FJ809750	♀	M	M	L	M	M	I	M	M	M	I	V	M	M	–
*Toxolasma parvum*	HM856639	♀^a^	M	V	L	M	M	M	I	M	M	I	V	M	I	I
*Uniomerus tetralasmus*	BK010480	♀	M	V	L	V	M	I	I	M	M	I	I	M	V	–
*Venustaconcha ellipsiformis*	FJ809753	♀	M	V	L	M	M	I	I	M	M	I	V	L	I	–
*Lampsilis powellii*	MF326972	♂	M	V	V	M	I	V	I	I	I	M	L	M	M	V
*Lampsilis siliquoidea*	MF326974	♂	M	V	V	M	I	V	M	I	M	M	L	M	M	V
*Potamilus alatus*	KU559010	♂	M	M	M	M	I	M	I	I	M	M	M	M	M	M
*Potamilus streckersoni*	ON881148	♂	M	M	M	M	I	M	M	I	M	M	M	M	M	M
*Quadrula quadrula*	FJ809751	♂	M	V	V	M	I	M	M	I	I	M	M	M	M	–
*Venustaconcha ellipsiformis*	FJ809752	♂	M	V	V	M	I	I	M	I	M	M	L	M	M	–

GenBank accession numbers and type are provided for each mitogenome. Dashes indicate a gene that was not annotated on the assembly.

^a^ Hermaphroditic mtDNA.

We investigated the relative amount of F- and M-type mtDNA in gonadal tissue using read depth, which showed a ratio of ∼1.5 M-type to F-type mtDNA (F-type mean coverage = 146, M-type mean coverage = 231). Relative read depth was similar across each mtDNA, other than a peak in the putative F-type mtDNA control region, which is likely due to an unassembled repetitive region (Figure 3). However, this ratio may vary based on sampling timing given M-type mtDNA is localized in sperm. The adult male specimen used in this study was collected in November, and given spermatogenesis occurs in the closely related species *P. alatus* in July–August (Haggerty and Garner 2000), the specimen was likely collected outside of spawning season. Male specimens collected during active spermatogenesis may exhibit increased dosage of M mtDNA in gonadal tissues. Resources presented in this study will allow for the development of assays (e.g. qPCR and ddPCR) to assess relative ratios of F and M type mtDNAs, which may be useful for future research in the phenology and mitochondrial physiology of *P. streckersoni*.

Similar to previous studies, our phylogenetic reconstruction resolved F- and M-type mitogenomes as monophyletic, while also generally resolves tribal relationships across Ambleminae (Figure 2; Huang et al. 2019; Pfeiffer et al. 2019). *Potamilus* was resolved as monophyletic in both the F- and M-type lineages, with *P. streckersoni* sister to *P. alatus* (Figure 2).

## Author contributions

RM-T – conceptualization, methodology, formal analysis, investigation, writing – original draft, visualization, and funding acquisition. CHS – conceptualization, methodology, formal analysis, resources, data curation, writing – review and editing, supervision, and funding acquisition.

## Data Availability

Mitogenome sequence data generated in this study are available on GenBank of NCBI at https://www.ncbi.nlm.nih.gov under the accession numbers ON855351 and ON881148. The associated BioProject and SRA numbers are PRJNA851076 and SRR19754570, respectively.
